# Clinical versus radiological method for adjusting rotational alignment during femoral shaft fractures intramedullary nailing and the malrotation impact on the functional outcomes: early results from a prospective cohort study

**DOI:** 10.1186/s13018-023-04300-8

**Published:** 2023-10-29

**Authors:** Ibrahim Mostafa Abbas, Ahmed A. Khalifa, Hossam Abubeih, Aly Mohamedean, Osama Farouk

**Affiliations:** 1 Orthopaedic Department, El-Eman General Hospital, Assiut, Egypt; 2 Orthopaedic Department, Qena Faculty of Medicine and University Hospital, South Valley University, Kilo 6 Qena-Safaga Highway, Qena, Egypt; 3 Orthopaedic Department, Faculty of Medicine, Assiut University Hospital, Assiut University, Assiut, Egypt

**Keywords:** Femoral shaft fracture, Intramedullary nailing, Femoral rotational malalignment, Functional outcomes, Radiolucent operative table

## Abstract

**Objectives:**

The primary objective of the current study is to assess which is better for obtaining the proper femoral rotation during IMN of femoral fractures, the radiological or clinical method. The secondary objectives were to document malrotation's incidence and its effect on the hip and knee functional outcomes.

**Methods:**

Thirty-three patients with unilateral femoral shaft fractures were treated using intramedullary nails (IMN) on a usual radiolucent operative table. Intraoperative rotation adjustment was performed using a radiological method (relying on the contralateral lesser trochanter profile) in 16 patients (group A), while in 17 patients, a clinical method was used (group B). Postoperative assessment of malrotation was performed using a CT scan, and 15 degrees was the cutoff value where below is an acceptable rotation (group I) and above is true malrotation (group II). Functional assessment was performed using the Harris hip score (HHS), the Tegner Lysholm Knee Scoring Scale (TLKSS), and the Neer score.

**Results:**

The patients' mean age was 30.7 ± 9.3 years; 81.8% were males, and the left side was injured in 63.6% of patients. After a mean follow up of 18.2 ± 6.9 months, all fractures were united, and the overall mean amount of rotational difference between the fractured and the contralateral side was 14.7° ± 6.0 (3–29.4), 84.8% were in external rotation. No difference in the mean rotational deformity in group A compared to group B. Measurements were 13.9 ± 6.7 and 15.7 ± 5.5, respectively (*p* = 0.47). Seventeen (51.5%) patients in group I with a mean deformity of 9.8 ± 3.4 (3–14.7), while group II consisted of 16 (48.5%) patients with a mean deformity of 19.6 ± 3.7 (15.3–29.4). There was no difference in the functional scores between group I and group II; HHS was 89.4 ± 7.4 versus 87.7 ± 8.9 (*p* = 0.54), TLKSS was 84.6 ± 9.6 versus 80.4 ± 13.9 (*p* = 0.32), and Neer score was 87.9 ± 9.5 versus 83 ± 12.5 (*p* = 0.21) for group I and group II, respectively.

**Conclusion:**

There was no difference in malrotation incidence after unilateral femoral fractures IMN with either an intraoperative clinical or radiological method for rotational adjustment; furthermore, malrotation did not affect the functional outcomes.

## Introduction

Treating femoral shaft fractures using intramedullary nailing (IMN) (either antegrade or retrograde) became the gold standard option in adult patients, owing to its relative safety, reproducibility, and ability to address fractures at different anatomical locations (peri trochanteric, subtrochanteric, shaft, and supracondylar fractures) [1–3]. This management option enabled surgeons to achieve high femoral fracture union rates, reaching up to 99% owing to its biological advantages (preserving the fracture hematoma), closed indirect fracture reduction, and minimal soft tissue dissection [3, 4].

IMN is usually performed as a closed maneuver, either with or without fluoroscopic control; however, it is prone to fracture malreduction with a resultant malalignment (in coronal, sagittal, and axial planes) and improper leg length restoration [5–7]. Rotational malalignment (malrotation in the axial plane) is by far the most frequent complication after femoral fractures IMN, reaching an incidence of up to 35%, which is usually challenging to detect intraoperatively using radiological images or postoperatively by clinical evaluation [8–11].

To avoid such complication, various methods were suggested for controlling the femoral rotation while performing IMN; most of these methods depend mainly on replicating the rotation profile of the contralateral intact side; however, there has yet to be an agreement on a single most accurate method [8]. Some clinical methods could be used, such as comparing the foot and hip joint rotation on both sides; however, this is only possible after securing the IMN in place, which showed a higher incidence of missed malrotation in more than 40% of the patients [8, 10, 12]. Radiological methods rely on copying the profile of the lesser trochanter from the intact contralateral side to the fractured side, which is widely used by most surgeons [10, 11, 13, 14]. Another method that is only helpful in cases with pure transverse fractures is to compare the cortical thickness above and below the fracture [15]. Furthermore, computer navigation-assisted surgery significantly reduced malrotation incidence; however, this technology is unavailable for most surgeons [16].

Malrotation after IMN could affect patients' outcomes and satisfaction after surgery [9, 17, 18]; however, the threshold point to consider a femoral fracture as malrotated or not when compared to the contralateral intact side is controversial among reports; some authors considered 10 degrees as the cutoff value, while others raised this to 15 degrees; this threshold differs according to the patient's ability to compensate for this deformity without symptoms [8, 10, 19], however, most surgeons agreed that malrotation greater than 30 degrees or affecting patients functional outcomes would need surgical correction [20, 21].

The primary objective of the current study was to assess which is better for obtaining the proper femoral rotation during IMN of unilateral femoral shaft fractures, the radiological or clinical method. The secondary objectives were to document malrotation's incidence and its effect on the hip and knee functional outcomes. We hypothesized that the radiological method is more accurate for obtaining the proper rotation, and if malrotation is present, it will negatively affect the functional outcomes.

## Patients and methods

After obtaining ethical committee approval *(IRB no. 17101876),* a prospective cohort study was carried out over three years starting from January 2018 to include patients presented with femoral shaft fractures and treated with IMN. During this period, we received 75 patients with femoral shaft fractures admitted to the trauma unit, Orthopaedic Department, El-Eman General Hospital, Assiut, Egypt.

We included adult patients with a recent (within 3 weeks) unilateral femoral shaft fracture (closed or open) who were amenable to IMN. Patients presented with bilateral femoral fractures, ipsilateral lower extremity skeletal injuries, segmental femoral shaft fractures; if the lesser trochanter on the same side was fractured, previous contralateral proximal femoral fracture resulted in anatomical distortion, and patients refused to participate in the study were excluded. Of the 75 patients presented during the study period, 38 were eligible, and five refused to participate, leaving 33 patients to be included, who were all available until the last follow up.

Upon admission to the hospital, initial assessment and management were carried out according to the ATLS protocol. For femoral fractures assessment, an anteroposterior (AP) and lateral plain radiographs were obtained (including the ipsilateral hip and knee). Fractures were classified for comminution according to the Winquist-Hansen classification and fracture geometry according to the AO/Orthopaedic Trauma Association classification systems.

*Operative details* The average time between the initial trauma and the surgical intervention was 2.1 ± 2.9 days (1–17); all patients were operated upon under spinal anesthesia, supine position, using a usual radiolucent operative table, where a C-arm fluoroscopy unit was on the opposite side. A reamed IMN with a piriformis entry point was used in all cases; either radiological or clinical assessment methods for rotational alignment adjustments as described in the literature were adopted [5, 8, 22, 23]; the method used was according to surgeon preference. *In the clinical method* First, we assess the hip range of motion (maximum internal and external rotation) of the uninjured side with the knee and hip flexed 90 degrees; we also note the foot position while the leg is resting on the table. During surgery, both sides are draped; after inserting the nail, one distal locking screw is inserted; the fractured side is now evaluated for hip range of motion, aiming at a hip rotation that matches the uninjured side, and if there is any adjustment heed to be made before final fixation. Also, we keep the whole limb in an extended position and compare the resting foot position with the other side. *In the radiological method,* the technique relying on the lesser trochanter profile is utilized. First, the contralateral uninjured limb is kept straight with the patella facing upward; an AP view of the hip is taken and stored, noting the shape of the lesser trochanter. After nail insertion and proximal locking in the fractured side, the distal part of the limb is rotated so that the patella is facing upward; then, the proximal fragment is rotated either internally or externally using the nail aiming jig till we get a lesser trochanter profile similar to the uninjured side.

Mini-open assisted reduction was required in 10 (30.3%) patients; all were from the clinical method group. Two (6%) patients had concomitant skeletal injuries (one fracture humerus and one fracture clavicle), which were treated at the same session by open reduction and internal fixation. According to the intraoperative technique used to adjust the femoral rotational alignment, patients were divided into two groups, wherein in group A (16 patients), a radiological method was used, and in group B (17 patients), rotational alignment was assessed clinically.

### Postoperative care and follow up protocol

After obtaining AP and lateral radiographs immediately postoperatively to ensure the quality of reduction and the implant positioning, patients were allowed to start active hip and knee mobilization under the supervision of a physiotherapist from the first postoperative day. Partial weight bearing was allowed according to the fracture configuration and the patients' competence. Patients were discharged from the hospital after a mean of 10.21 ± 1.12 days (3–20).

Follow up visits were scheduled at two weeks (for wound check and stitches removal), at six weeks for initial radiographic evaluation of the union process, and to determine the weight-bearing status. Then at three, six,12 months, and then annually. The fracture was considered united if there was an evident bridging callus in both AP and lateral plain radiographs (2–3 cortices) and clinically by the patient's ability to pain-free weight bearing.

**Fig. 1:**
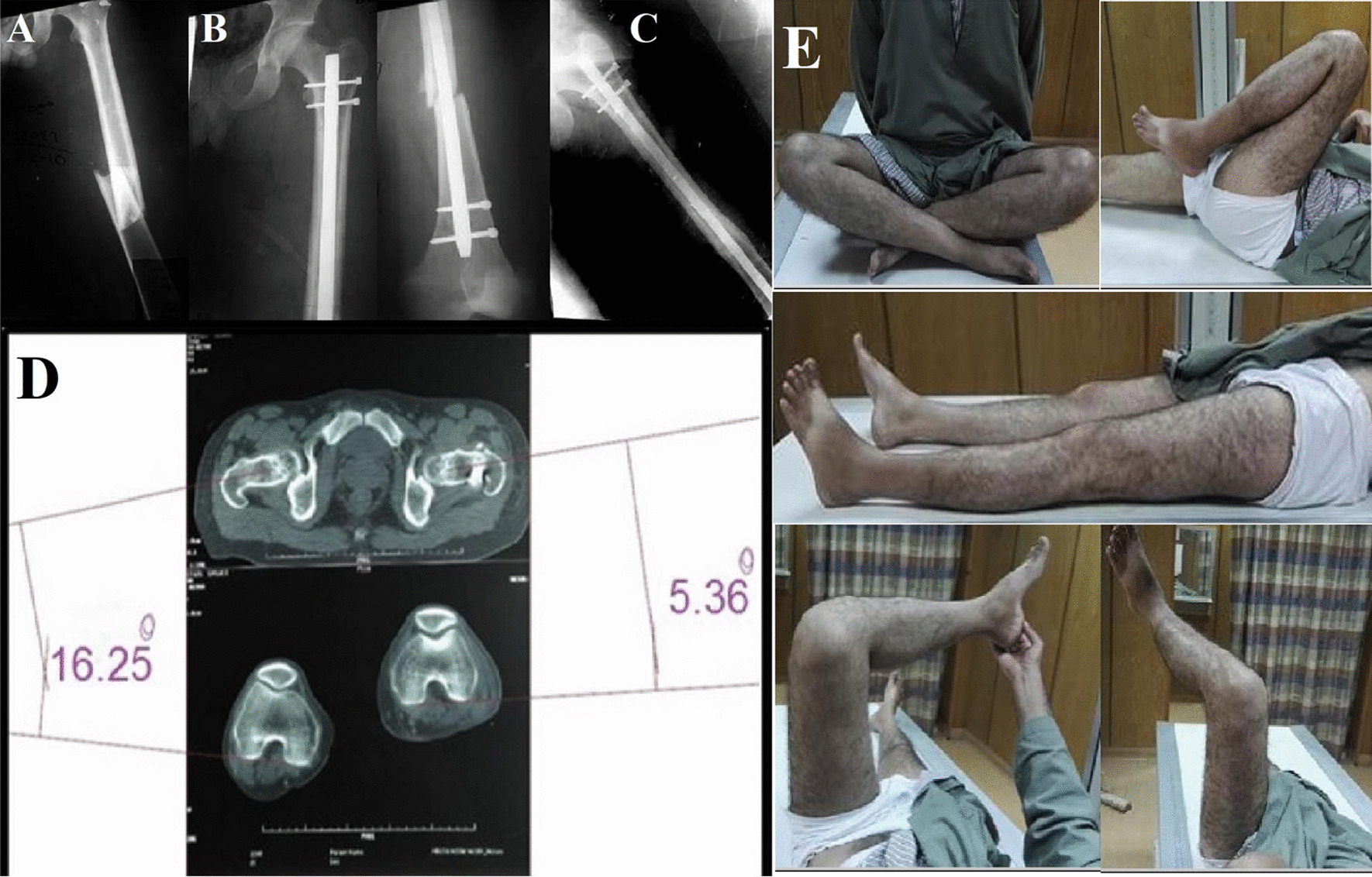
Male patient, 32 years old, sustained a left femur fracture, he was treated by IMN, and the rotation was adjusted using the clinical method (Group B). **A** preoperative radiographs. **B** postoperative radiographs. **C** follow up radiographs at 14 months follow up showing complete union of the fracture. **D** rotational profile measured using CT scan, showing external rotation deformity of the left side of 21.6 degrees (Group II) compared to the intact right side. **E** clinical images showing the functional outcomes

**Fig. 2:**
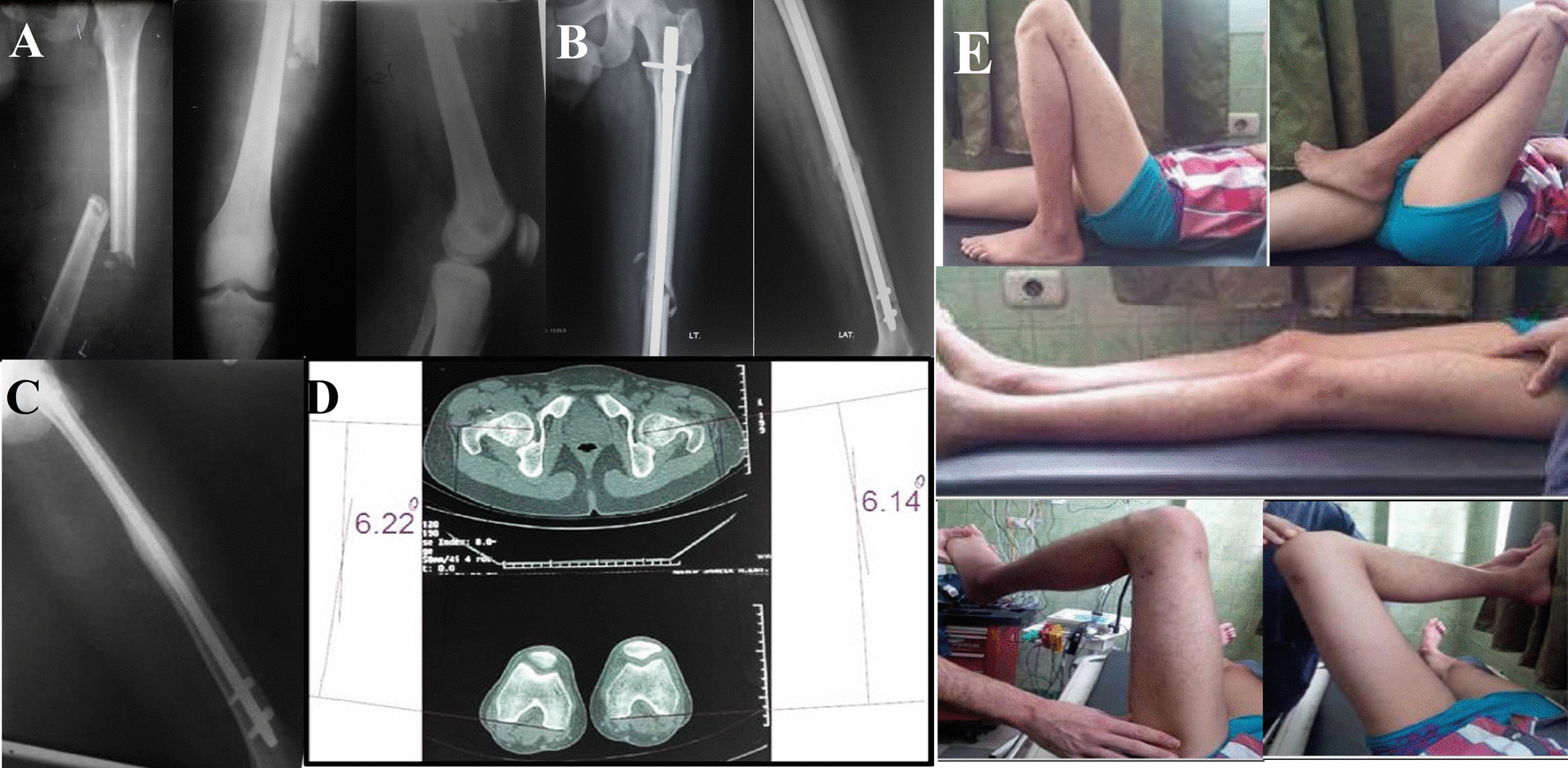
Male patient, 26 years old, sustained a left femur fracture, he was treated by IMN, and the rotation was adjusted using the radiological method (Group A). **A** preoperative radiographs. **B** postoperative radiographs. **C** follow up radiographs at 12 months follow up showing the complete union of the fracture. **D** rotational profile measured using CT scan, showing external rotation deformity of the left side of 12.4 degrees (Group I) compared to the intact right side. **E** clinical images showing the functional outcomes

At the last follow up, all patients were assessed clinically using the Harris hip score (HHS), the Tegner Lysholm Knee Scoring Scale (TLKSS), and the Neer score [24]. The rotational alignment of the lower limb was assessed by obtaining a CT scan for both lower limbs using the method described by Jeanmart et al. [25]; then, patients were further divided into two groups according to the presence of significant malrotation, considering 15 degrees as the cutoff value [10, 19, 26], group I included patients with accepted rotation (< 15 degrees), and group II included patients with true malrotation (≥ 15 degrees) (Figs. 1 and 2).

### Statistical analysis

Statistical analysis was performed using IBM SPSS Statistics Version 23. continuous variables described by mean and standard deviation, and categorical variables were described by numbers and percentages. Student's *t* tests were used to compare continuous variables. The chi-square test and Fisher exact test were used to compare categorical variables. The correlation between different functional scores with the amount of rotation was assessed by Pearson correlation. A *p* < 0.05 was considered statistically significant.

## Results

The patients' mean age was 30.7 ± 9.3 years (range from 20 to 63); 27 (81.8%) were males, the left side was injured in 21 (63.6%) patients, the mean BMI was 25.1 ± 3.1 (range from 18.5 to 30, 16 (48.5%) were normal weight, 13 (39.4%) were overweight, and four (12.1) were obese) and most of the cases were injured during a road traffic accident, 25 (75.8%) patients. Fractures were classified into 15 (45.4%) type I, 9 (27.3%) type II, 6 (18.2%) type III, and 3 (9.1%) type IV, according to Winquist Classification. While according to AO classification, 13 fractures (39.3%) were type A, 17 fractures (51.5%) were type B, and three fractures (9%) were type C. Six (18.2%) fractures were open ( two type I, and four type II, according to Gustillo classification). Patients’ details are shown in (Table 1).

**Table 1:** Patients, trauma, and fracture characteristics

Variables		Group A (n = 16)	Group B (n = 17)	*p* value
Age*		26.6 ± 5.2	33.9 ± 10.9	**0.02**
Gender**	Male	12 (75%)	11 (64.7%)	0.47
	Female	4 (25%)	6 (35.3%)	
Side**	Right	11 (68.7%)	10 (68.8%)	0.55
	Left	5 (31.3%)	7 (41.2%)	
BMI*		25.3 ± 3.3	24.8 ± 3	0.72
Mechanism of injury**	Animal Kick	0 (0.0%)	1 (5.9%)	**0.04**
	FAI	0 (0.0%)	3 (17.6%)	
	FFH	3 (18.7%)	2 (11.8%)	
	FOG	2 (12.5%)	2 (11.8%)	
	RTA	11 (68.8%)	9 (52.9%)	
Fracture classification				
Winquist-Hansen**	Type I	8 (50.0%)	7 (41.3%)	0.37
	Type II	5 (31.2%)	4 (23.5%)	
	Type III	3 (18.8%)	3 (17.6%)	
	Type IV	0 (0.0%)	3 (17.6%)	
AO-OTA**	A2	0 (0.0%)	3 (17.6%)	0.13
	A3	8 (50.0%)	2 (11.8%)	
	B1	1 (6.3%)	1 (5.9%)	
	B2	5 (31.3%)	5 (29.4%)	
	B3	2 (12.5%)	3 (17.6%)	
	C1	0 (0.0%)	1 (5.9%)	

Group A:rotation was adjusted by radiological method, Group B: rotation was adjusted by clinical method

*Data presented as mean ± SD, **Data presented as number (percentage). Significant values are presented in bold

BMI: body mass index, FAI: firearm injury, FFH: fall from a height, FOG: fall on ground, RTA: road traffic accident

After a mean follow up of 18.2 ± 6.9 months (8–34), all patients were available for assessment, and all fractures were united. The overall mean amount of rotational difference in the fractured side compared to the contralateral intact side was 14.7° ± 6.0 (3–29.4); the deformity was external rotation in 28 (84.8%) patients, while five (15.2%) had an internal rotation deformity. The mean rotational deformity in group A compared to group B was 13.9° ± 6.7 and 15.7° ± 5.5, respectively, with no difference between both groups (*p* = 0.47). Seventeen (51.5%) patients belong to group I with a mean deformity of 9.8° ± 3.4 (3–14.7), while group II consisted of 16 (48.5%) patients with a mean deformity of 19.6° ± 3.7 (15.3–29.4) (all were in external rotation). Five (50%) out of ten patients who had a mini-open assisted fracture reduction had a true rotational deformity, compared to 11 (47.8%) of the patients who had closed reduction; the difference was insignificant (*p* value = 0.45).

 Regarding the functional scores, for the three measured scores, there was no difference between patients who had an accepted rotation (group I) and those with true malrotation (group II), and the overall scores were as follows, HHS was 89.4 ± 7.4 (100–74) versus 87.7 ± 8.9 (100–70) (*p* = 0.54), TLKSS was 84.6 ± 9.6 (100–71) versus 80.4 ± 13.9 (100–57) (*p* = 0.32), and Neer score was 87.9 ± 9.5 (100–64) versus 83 ± 12.5 (100–60) (*p* = 0.21) for group I and group II, respectively. Details of the score’s classes are presented in (Table 2). Furthermore, there was an insignificant correlation between the amount of deformity and the functional scores, HHS (r = − 0.21, *p* = 0.23), TLKSS (r = − 0.18, *p* = 0.31), and Neer score (r = − 0.15, *p* = 0.40).

**Table 2:** Differences between functional scores classes

	Group I (n = 17)		Group II (n = 16)	*p* value
Harris hip score				
*Mean ± SD*	89.4 ± 7.4		87.7 ± 8.9	0.54
Class				0.69
Excellent	9 (52.9%)		6 (37.5%)	
Good	7 (41.2%)		7 (43.8%)	
Fair	1 (5.9%)		2 (12.5%)	
Poor	0		1 (6.3%)	
Lysholm knee score				
*Mean ± SD*	84.6 ± 9.6		80.4 ± 13.9	0.32
Class				0.55
Excellent	5 (29.4%)		4 (25%)	
Good	9 (52.9%)		5 (31.3%)	
Fair	3 (17.6%)		4 (25%)	
Poor	0		3 (18.8%)	
Neer score				
*Mean ± SD*		87.9 ± 9.5	83 ± 12.5	0.21
Class				0.29
Excellent		12 (70.6%)	6 (37.5%)	
Satisfactory		4 (23.5%)	8 (50%)	
Unsatisfactory		1 (5.9%)	2 (12.5%)	

Group I (accepted rotation), Group II (true malrotation)

## Discussion

We found that there was no difference regarding the incidence of malrotation deformity after IMN of unilateral femoral fractures operated on a usual radiolucent table either after using an intraoperative clinical or radiological method for rotational adjustment; furthermore, the functional outcomes were not affected by the presence or the degree of malrotation, so both our hypotheses were disputed.

Malrotation is considered one of the most common complications when treating femoral shaft fractures using IMN [8, 9]. Various factors could lead to this problem, some of which are related to the characteristics of the fracture (such as configuration and comminution), some could be related to the surgical technique (such as using fracture table and patient position), and patient factors (such as operating on obese patients) [5, 8, 27, 28]. Moreover, the direction and the amount of malrotation are determined mainly by the fracture location and the muscles acting around the fracture [5]. The malrotation incidence documented in the literature varied among studies and was reported to occur in 35% to 42% of the patients [8–11, 17]. In the current study, we reported an incidence of malrotation of 48.5%, which is slightly higher than what is reported in the literature. Furthermore, most of the deformities (84.8%) were in external rotation, although we did not investigate the predisposing factors; this could be attributed to the muscle forces working around the fracture, about 52% of the included patients were obese or overweight, operating in a supine position, and not using a traction table [5, 6, 23]. However, we cannot deny possible surgical technique issues predisposing to the deformity, specifically using manual traction to maintain the reduction till final fixation [5, 8].

Suggested advantages of using a fracture table over a regular radiolucent one are the less assistance needed and maintenance of the fracture position while operating [28, 29]; however, it carries some complications such as nerve palsies, perineal soft tissue injuries, ankle joint pain, and possible increased risk of femoral internal malrotation [30]. We were comfortable operating all cases on the usual radiolucent operative tables while the patient was supine, using manual traction to assist fracture reduction. Some studies showed no difference in malrotation incidence according to the operative table type, as shown in a randomized study by Rashid et al. included 74 patients having femoral fractures treated with IMN; 37 were operated upon using a fracture table and 37 on a regular operative table, and the overall incidence of malrotation was 17.6% (76.9% of them were internally rotated), which was not different between both groups (*p* = 0.760) [29]. However, on the contrary, in a randomized study by Stephen et al. operating using a fracture traction table (42 patients) versus manual traction (45 patients), the authors reported significantly more internal malrotation (> 10 degrees) in patients operated on traction table compared to manual traction, 29% versus 7%, respectively (*p* = 0.007). Furthermore, they reported lower mean operative time with the manual traction technique than operating on a traction table, 119 versus 139 min, respectively (*p* = 0.033) [28].

Several techniques were described for obtaining proper rotation during IMN of femoral shaft fractures, including direct visualization (if an open reduction was performed), morphological fracture characteristics, and fracture reduction alignment under fluoroscopy [8, 15, 27, 31]. Furthermore, different techniques relying on using the uninjured contralateral side (either radiologically or clinically) as a template have been used by many surgeons [11, 22, 26]. In the current study, we did not encounter a difference in the incidence of malrotation after either the radiological or clinical method relying on the normal contralateral side. On the contrary, a study by Deshmukh et al. compared a radiological method while operating on a fracture table (by using the profile of the lesser trochanter on the intact side as a template) to a conventional clinical technique; the mean malrotation was 12.5° (range 6.4–17.7) with the clinical technique compared to 4.1° (range 0–9.9) with the radiological technique, the difference was significant (*p* = 0.016) [22]. In a study by Mansouri-Tehrani et al., who treated 140 patients with isolated femoral fractures using IMN over six years, intraoperative assessment of rotational profile was performed by clinical method, postoperative malrotation assessment using a CT scan showed a 15.7% incidence of malrotation of 10–15 degrees [32].

The caveat of using the contralateral side as a template originated from the possibility of deformity or version difference in the supposed normal contralateral side compared to the injured side [27, 33]. In a CT-based study by Croom et al. to evaluate the difference in femoral version between both sides in uninjured individuals, they included 164 subjects, the mean version difference between both sides was 5.4° ± 4.4, 17.7% had a difference in version ≥ 10 degrees, and 4.3% had a difference in version ≥ 15 degrees. They concluded that a possible difference in the femoral version between both sides could be present and proposed a 15 degrees difference as the point for considering malrotation [27].

We assessed the rotational profile by CT scan images; however, various methods, including clinical and radiological, for postoperative malrotation assessment were described, but both are unreliable and less accurate [8]; however, most surgeons rely on assessing the rotational profile by obtaining a CT scan of both sides, which proved to be an accurate and efficient method [34, 35]. We adopted the technique described by Jeanmart et al. [25], where the angle was calculated between the femoral neck axis and a line tangential to the posterior condyles, which was also utilized in the study by Karaman et al. [9]; however, various techniques using various lines were described [26, 27].

How much malrotation a patient can tolerate and compensate for is controversial among authors; most surgeons agreed that malrotation less than 10 degrees will pass unnoticed by the patient and easily compensated as it is considered within the normal variations limit, while malrotation above 15 degrees will be considered as pathological malrotation, and malrotation between 10 and 14 degrees is a grey or controversial zone [5, 8]. Some authors, such as Kent et al., raised the acceptance range of malrotation between 15 and 30 degrees, believing that most patients could tolerate this deformity amount [21].

The patient compensates for the malrotation by rotating the lumbosacral spine, hip joint, knee joint, and up to the foot and ankle; these compensatory mechanisms are more evident while walking [36]. However, If the malrotation was large enough, it could be noted by the patients and cause cosmetic disfigurement; furthermore, it could negatively affect these compensatory mechanisms, leading to lower hip and knee functional outcomes, less patient satisfaction, and patients' daily activities affection [8, 9, 17, 18].

Although we had a relatively high incidence of malrotation compared to the literature, we achieved excellent or good functional outcomes in the hip and knee joints in 87.9% and 96.7% of the patients, respectively. Furthermore, according to Neer's score, excellent or satisfactory results were obtained in 90.9% of the patients, and the presence and the degree of malrotation did not affect the functional outcomes.

The effect of femoral malrotation on functional outcomes differs among studies as it is affected by patients' ability to compensate for the deformity; in a study by Bråten et al., although malrotation of more than 15 degrees was reported in 21 patients, only eight had a clinical complaint [37]. In a study by Gugala et al. on 16 patients, they reported internal rotation deformity (3–13 degrees) in five patients, while in 11 patients, an external rotation deformity (3–32 degrees) was evident, the authors reported no difference regarding patients satisfaction according to the deformity direction; however, external rotation deformity was better tolerated compared to internal rotation [38]. Furthermore, Mansouri-Tehrani et al. reported no correlation between femoral malrotation and clinical outcomes [32].

On the contrary, many authors reported that femoral malrotation affects functional outcomes, especially its negative effect on the patellofemoral joint, resulting in persistent anterior knee pain [10, 18, 26]. In a study by Karaman et al. on 24 patients who were treated by IMN for a unilateral femoral fracture, they reported an incidence of malrotation of about 42% as compared to the contralateral intact side; they reported lower TLKSS and WOMAC scores (for the knee and the hip joints) in patients with malrotation (no difference between external or internal deformity) compared to those who did not have deformity, patients complained mainly of anterior knee pain while climbing stairs and performing sports activities; furthermore, some patients in the malrotation group reported occasional hip pain [9].

We admit that the current study has some inherent limitations. First, we did not perform a sample size calculation before the study; however, we believe we included enough average number of patients compared to the previously published studies, enabling us to obtain sensible results. However, we admit that a larger sample-size study would be preferable. Second, is the lack of randomization. Third, we did not calculate the amount of radiation exposure from performing a CT scan to assess the rotational profile; however, according to the radiology department, the radiation was kept to a minimum within the range of accepted limits [39]. Last, the relatively short follow up period, as a longer follow up is needed to estimate the possible long-term effects of malrotation on the outcomes.

## Conclusions

There was no difference in the amount of femoral malrotation after treating a unilateral femoral shaft fracture using IMN, either after utilizing intraoperative clinical or radiological methods for obtaining proper rotation. The functional outcomes were accepted in most patients, which did not differ between patients with an accepted rotation or true malrotation. Further well-designed randomized studies will help support our findings.

## Data Availability

All the data related to the study are mentioned within the manuscript; however, the raw data are available with the corresponding author and will be provided upon a written request.

## Abbreviations

IMN: Intramedullary nailing
AP: Anteroposterior
HHS: Harris hip score
TLKSS: Tegner Lysholm Knee Scoring Scale
